# Effects of* Angelica dahurica* and* Rheum officinale* Extracts on Excisional Wound Healing in Rats

**DOI:** 10.1155/2017/1583031

**Published:** 2017-08-16

**Authors:** Wan-Ting Yang, Chun-Yen Ke, Wen-Tien Wu, Horng-Jyh Harn, Yi-Hsiung Tseng, Ru-Ping Lee

**Affiliations:** ^1^Institute of Medical Sciences, Tzu Chi University, Hualien 97004, Taiwan; ^2^Department of Nursing, St. Mary's Medicine Nursing and Management College, Yilan 266, Taiwan; ^3^Department of Nursing, Tzu Chi University of Science and Technology, Hualien 970, Taiwan; ^4^Department of Orthopedics, Hualien Tzu Chi Hospital, Buddhist Tzu Chi Medical Foundation, Hualien 97002, Taiwan; ^5^Department of Pathology, Hualien Tzu Chi Hospital, Buddhist Tzu Chi Medical Foundation, Hualien 97002, Taiwan; ^6^Institute of Molecular Biology, National Chung Hsing University, Taichung 402, Taiwan

## Abstract

The main objective of wound treatments is to restore the functional skin properties and prevent infection. Traditional Chinese medicine provides alternative anti-inflammatory, antimicrobial, and wound healing therapies. Both* Angelica dahurica *extract (AE) and* Rheum officinale* extract (RE) possess antimicrobial activity. In this study, AE and RE were applied in wound treatment to investigate their healing effects. Thirty Sprague-Dawley rats with dorsal full-thickness skin excision were divided into normal saline (NS), AE, RE, AE plus RE (ARE), and Biomycin (BM) groups. The treatment and area measurement of wounds were applied daily for 21 days. Wound biopsies and blood samples were obtained for histology examinations and cytokine analysis. Results showed that wound contraction in ARE group was significantly higher than that in NS and BM groups (*P* < 0.05). Histological analysis showed that more inflammatory cell infiltration, collagen fibers, and myofibroblasts were observed in ARE treated group than those in NS group on days 3–5. In ARE group, plasma IL-6 levels were elevated during days 3–5 (*P* > 0.05), and plasma TGF-*β*1 levels were significantly lower than those in the NS group on days 3-4 (*P* < 0.05). In conclusion, ARE accelerates wound healing during inflammation and proliferation phases.

## 1. Introduction

The skin, the largest organ of the body, forms an indispensable barrier that protects against injury and infection [1]. When injury occurs, a complex cascade of biological processes begins to repair and regenerate the damaged tissue. The wound healing process comprises four continuous and overlapping phases: hemostasis, inflammation, proliferation, and remodeling [2]. In general, the goals of wound care and treatment are to prevent infection, reduce swelling and inflammation, accelerate wound healing, and minimize scar formation [3]. Antimicrobial agents such as ciprofloxacin, mupirocin, and neomycin sulfate are commonly used to prevent and treat infections [4], while corticosteroids and nonsteroidal anti-inflammatory drugs are used to reduce swelling and inflammation of the skin [5]. Some supplements, such as vitamin E, glutamine, zinc, and freshwater clam extract, have also been shown to be effective in wound healing [6, 7].

In recent years, traditional Chinese medicine (TCM) and natural compounds obtained from a variety of herbs have become popular as alternative and complementary therapies [8]. Moreover, numerous studies have demonstrated the effectiveness of extracts from plants such as* Aloe vera*,* Calendula officinalis*,* Abrus cantoniensis*, and* Ampelopsis japonica *in treating skin lesions [9–11]. Compared with the common use of therapies, such as antimicrobial agents and anti-inflammatory drugs, the advantage of using TCM therapy for skin lesions is that it may have multiple active effects, such as anti-inflammatory activity, antimicrobial activity, cell stimulation, and the promotion of wound healing [9]. This study therefore investigated the treatment of skin wounds with TCM extracts.* Rheum officinale (da huang)* and* Angelica dahurica (bai zhi)*, which have heat-clearing, toxin-resolving, swelling reduction, anti-inflammatory, antimicrobial, and antibacterial properties, were chosen for the wound treatment [12–18]. Emodin, an active compound in* R. officinale*, has been shown to improve wound healing in rats [19]. Furthermore,* A. dahurica* extract has been demonstrated to accelerate the proliferation of rat skin cells and human keratinocytes* in vitro* [20, 21]. However, using* A. dahurica* or/and* R. officinale *on treating wound has not been studied yet. In this study, an excisional wound rat model was exploited using extracts prepared from these two herbs to investigate their effects on wound healing.

## 2. Materials and Methods

### 2.1. Preparation of the TCM Extracts

*A. dahurica* and* R. officinale* powders were purchased from Hou-Chuia Biopharm Co., Ltd. Tainan City, Taiwan. Ethanol extracts of these TCMs were prepared separately by adding 50 g of the powders into 400 mL of 70% ethanol and heating the mixtures at 70°C for 24 h. The mixtures were then centrifuged at 10,000 ×g for 15 min and the supernatants were concentrated under reduced pressure to remove the ethanol. Equal volumes of* A. dahurica* extract (AE) and* R. officinale* extract (RE) were mixed and designated as ARE. The AE, RE, and ARE were sterilized by autoclave before application.

### 2.2. Antimicrobial Susceptibility Test of the TCM Extracts

Disk diffusion test was performed according to CLSI standards [22] to test the antimicrobial susceptibility of AE, RE, and ARE.* S. aureus *ATCC 29213 (Bioresource Collection and Research Center, BCRC, Hsinchu, Taiwan) was cultured in Tryptic Soy Broth (Becton, Dickinson and Company, USA) at 37°C until log phase and the turbidity was adjusted with the broth to achieve 0.5 McFarland standard (approximately 10^8^ CFU/mL). The autoclaved 6 mm diameter filter paper disks (Advantec Grade Number 1) were soaked with 20 *μ*L of AE, RE, or ARE and dried at room temperature. The inoculums (~10^7^ cells) were spread onto Mueller-Hinton agar (Becton, Dickinson and Company, USA), and then the disks were placed onto the agar. Disks containing distilled water were used as negative control (Blank). The results were expressed as the means ± SE of diameters (mm) of the inhibition zone scored after overnight incubation.

### 2.3. Anti-Inflammatory Test of the TCM Extracts

Human acute monocytic leukemia (THP-1) cells (BCRC, Hsinchu, Taiwan) were cultured in RPMI-1640 medium (Gibco BRL, Gaithersburg, MD, USA) supplemented with 0.05 mM *β*-mercaptoethanol (Sigma-Aldrich, St. Louis, MO, USA), 2 g/L sodium bicarbonate, 1% penicillin/streptomycin (PS, Biological Industries, Beithemeek, Israel), and 10% fetal bovine serum (Gibco BRL, Gaithersburg, MD, USA) in a humidified atmosphere with 5% CO_2_ at 37°C. For anti-inflammatory test, THP-1 cells were seeded into 24-well culture plates at 1 × 10^5^ cells/mL/well, and then 1 *μ*g/mL lipopolysaccharides (LPS) (Sigma-Aldrich, St. Louis, MO, USA) were added to the wells to induce inflammation. The TCM extracts, AE, RE, or ARE (final concentration = 0.2%), were then added to the wells. After 24 h of coincubation, the supernatants were obtained by centrifuged at 15,000 ×g for 15 min and assayed using human TNF-*α* enzyme-linked immunosorbent assay (ELISA) kits (R&D System, MN, USA).

### 2.4. Animals

Thirty healthy male Sprague-Dawley (SD) rats weighing between 250 and 300 g were used in this study. The rats were placed in standard cages and housed in a humidity- and temperature-controlled room (55 ± 15%; 22 ± 1°C) with 12-h light-dark cycles. All the rats received standard amounts of food and water. All experiments were performed according to the institutional protocols of the Animal Usage Regulation Committee of Tzu Chi University (number 104084).

### 2.5. Excisional Wound Model and Treatment

The rats were anesthetized through ether inhalation for approximately 10 minutes, following which the back hair of the rats was shaved and a full-thickness excisional skin wound (area of 20 × 10 mm^2^, depth of 2 mm) was created on the dorsum of each rat by using forceps and scissors under sterile conditions [7]. After wounding, the rats were housed individually in clean cages and randomly classified into five groups: normal saline (NS), AE, RE, ARE, and Biomycin (BM). Treatments were administered once a day for 21 days. Wounds were cleaned gently with sterile NS swabs before treatment. In the NS group, no other treatment was provided after NS cleaning. In the AE, RE, and ARE groups, treatment involved the topical application of approximately 1 *μ*L/mm^2^ of TCM extract. The TCM extracts were kept on the wound. In the BM group, a thin layer of Biomycin ointment (CBC Biotechnological & Pharmaceutical Co., Ltd. New Taipei City, Taiwan) was administered using sterile swabs. After treatment, the wounds were left at air without covered, and the rats were housed individually in clean cages. The rats were sacrificed on days 3, 4, 5, and 21 after wounding, and wound biopsies and blood samples were obtained for analysis.

### 2.6. Wound Measurement

The wounds and a ruler with 1-mm increments placed beside the wounds were photographed with a digital camera (Nikon D70, Tokyo, Japan) once a day before treatment. The wound areas were measured using ImageJ (National Institutes of Health, United States). Percentage of wound area after contraction was calculated as follows: [1 − (*D*_0_ − *D*_*n*_)/*D*_0_] × 100% (*D*_0_, the proportion of wound area on day 0; *D*_*n*_, the proportion of remaining wound area on day *n*).

### 2.7. Histology Analysis

Cross-sectional full-thickness skin biopsies were obtained from the wound sites at days 3, 4, 5, and 21 after wounding for histological study. These wound samples were fixed with 4% buffered formaldehyde, embedded in paraffin, and divided into 5-*µ*m sections. The tissue sections were stained with hematoxylin and eosin (H&E) for general morphology and Masson's trichrome for collagen fibers observation. Immunohistochemistry (IHC) was performed using alpha-smooth muscle actin (*α*-SMA) antibodies for myofibroblasts observation. The tissue sections were deparaffinized, rehydrated, and treated with peroxidase reagent for 20 min at room temperature. The sections were then boiled with the citrate buffer (pH 6.0) for 30 min and blocked with the ImmunoBlock for 2 h at room temperature. After washing with PBS, the tissue sections were incubated with *α*-SMA antibodies (1 : 200 dilution; Abcam, Cambridge, MA, USA) for 3 h and then treated with mouse probe HRP labeling (BioTnA Inc., Kaohsiung, Taiwan) for 30 min at room temperature. The sections were stained with DAB brown (1 : 50 dilution; BioTnA) and counterstained with hematoxylin. After dehydration, sections on slides were covered with coverslips, and the results were analyzed using microscope.

### 2.8. Analysis of Cytokines

Blood samples were collected at days 3, 4, and 5 after wounding and centrifuged at 3000 ×g for 10 min at 4°C. The plasma samples were stored at −20°C for later measurement of cytokine concentration. The levels of inflammatory cytokine IL-6 and the angiogenesis factor TGF-*β*1 were measured using rat IL-6 and TGF-*β*1 ELISA kits (R&D Systems, MN, USA). Measurements were performed according to the manufacturer instructions. The optical density of each reaction was determined using a Dynex MRX II microplate reader (Chantilly, VA, USA) at 450 nm and converted into a concentration through the standard curve. The results were expressed as pg/mL.

### 2.9. Statistical Analysis

All values were expressed as mean ± standard error of the mean. Statistical analyses were performed through a *t*-test with IBM® SPSS® Statistics (version 22.0, SPSS Inc., IBM, Chicago, IL, USA). *P* values < 0.05 were considered statistically significant.

## 3. Results

### 3.1. Antimicrobial and Anti-Inflammatory Effects of AE and RE

**Figure 1:**
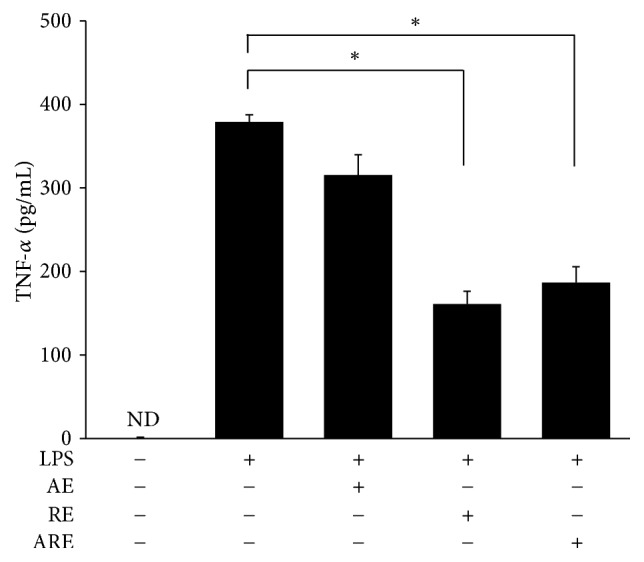
Anti-inflammatory effect of AE, RE, and ARE. Human acute monocytic leukemia (THP-1) cells stimulated by LPS were treated with AE, RE, and ARE. The amounts of TNF-*α* in the supernatants were measured by the enzyme-linked immunosorbent assay (ELISA). Data are expressed as mean ± standard error of the mean; ND means not detected. ^*∗*^*P* < 0.05 indicates RE group and ARE group compared with LPS group.

All of the AE, RE, and ARE have showed the antimicrobial activity against* S. aureu*s ATCC 29213 (Table 1). Furthermore, AE, RE, and ARE reduced the expression of TNF-*α* released by LPS-stimulated THP-1 cells, indicating the anti-inflammatory effects of the TCM extracts (Figure 1). These results revealed that these TCM extracts have antimicrobial and anti-inflammatory activities.

### 3.2. Effects of AE and RE on Wound Contraction

**Figure 2:**
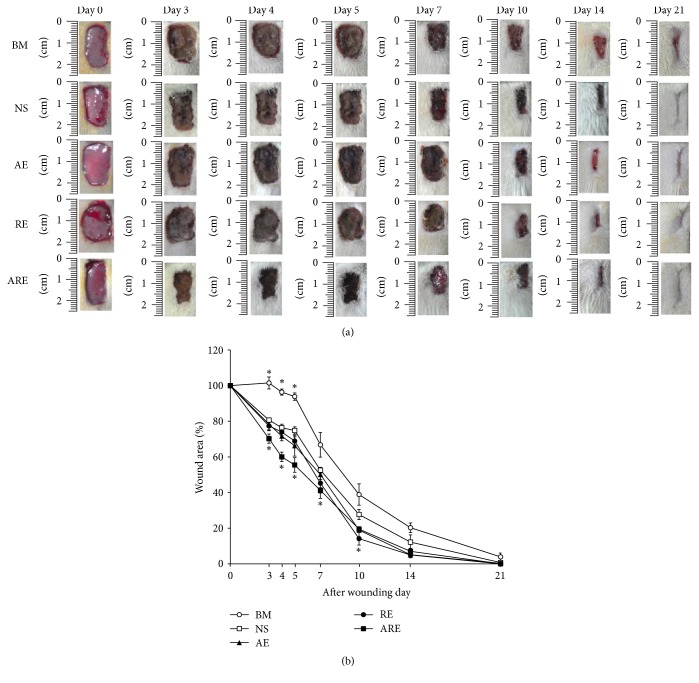
Macroscopic analysis of the wound healing process. (a) Macroscopic view of wounds treated with normal saline (NS), Biomycin ointment (BM),* Angelica dahurica *extract (AE),* Rheum officinale* extract (RE), and AE plus RE (ARE) on days 0, 3, 4, 5, 7, 10, 14, and 21. (b) Changes in wound sizes from day 0 to day 21, expressed as a percentage of the initial wound area. Data are expressed as mean ± standard error of the mean. ^*∗*^*P* < 0.05 indicates AE, RE, ARE, or BM group compared with NS group.

The AE, RE, and ARE were topical application on the wound site for treatment. The percentage of wound area after contraction represented the effects of each treatment on wound healing.  Figure 2(a) shows the photographs of wound sites on days 0, 3, 4, 5, 7, 10, 14, and 21 after wounding; Figure 2(b) shows the percentage of wound area after contraction measured using ImageJ from days 0 to 21. On days 3–10, the percentage of wound contraction in the ARE group was significantly lower than that in the NS group (Figure 2). The percentage of wound area after contraction in the ARE group was lower than that in the NS group on day 3 (70.25% versus 80.63%), day 4 (60.05% versus 76.41%), day 5 (55.16% versus 74.80%), day 7 (41.23% versus 52.61%), day 10 (19.52% versus 27.67%), and day 14 (12.23% versus 7.07%) (*P* < 0.05). On day 21, the wounds had nearly recovered. The wound areas of the ARE and NS group remained at 0.05% and 0.78%, respectively, and the percentages of wound contraction in the AE and RE groups did not differ significantly from that in the NS group (Figure 2(b)). However, the wound contraction of the BM group was less favorable than those of the other groups (Figure 2(b)). The histological characterization of the wound sites on day 21 showed almost complete healing on NS, AE, RE, and ARE groups, while scabs were still observed above the epidermis on the BM group.

### 3.3. Histological Observation of Healed Wound Area

**Figure 3:**
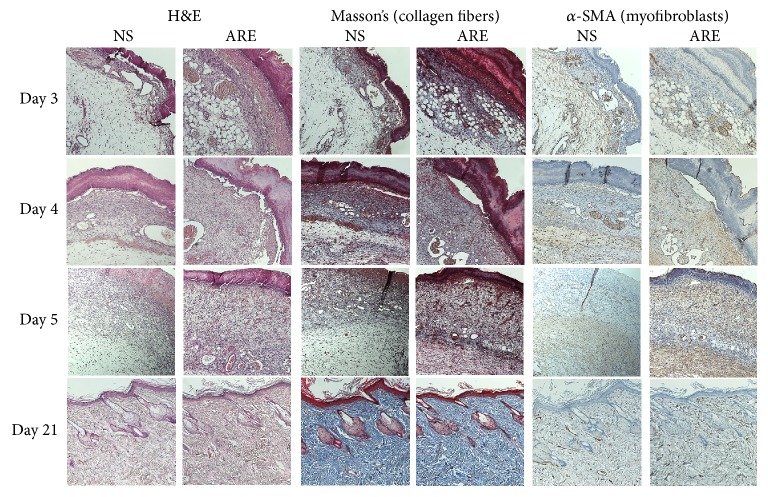
Histological view of skin wound on days 3, 4, 5, and 21, treated with NS and ARE. Stained with hematoxylin and eosin (H&E), Masson's trichrome, and alpha-smooth muscle actin (*α*-SMA) immunohistochemical (IHC) staining, at ×100 magnification.

Skin biopsies from the wound sites on days 3–5 were investigated to determine the treatment effectiveness. The histological observations showed that scabs and granulation tissues were observed at the wound sites, and more inflammatory cells were observed in ARE groups than NS group on days 3–5 (Figure 3). On day 3, H&E stain showed that the scabs in ARE group were thicker than those in NS group, and the dermis was loose in NS group while an organized formation in the dermis was observed in ARE group. Masson's trichrome stain showed that newly formed collagen fibers were observed in the dermis, and the presence of collagen fibers in ARE group was more than those in NS group (Figure 3). On day 4, the dermis in NS group was still loose, and the granulation tissues in ARE group were more than those in NS group. On day 5, the scabs in ARE group were thinner than those in NS group, and more myofibroblasts were observed in ARE group than NS group as shown in IHC of *α*-SMA (Figure 3). The histological characterization on day 21 of NS and ARE groups showed that new epidermis was constructed; hair follicles and dense irregular connective tissue were observed in dermis from H&E stain. In both groups, abundant and well-organized collagen were present in the dermis as shown in Masson's trichrome stain; myofibroblasts were absent in the dermis as shown in IHC of *α*-SMA. After 21 days of wound healing process, the wounds were almost healed and formed a complete structure of normal skin (Figure 3).

### 3.4. IL-6 and TGF-*β*1 Levels on Day 3 to Day 5

**Figure 4:**
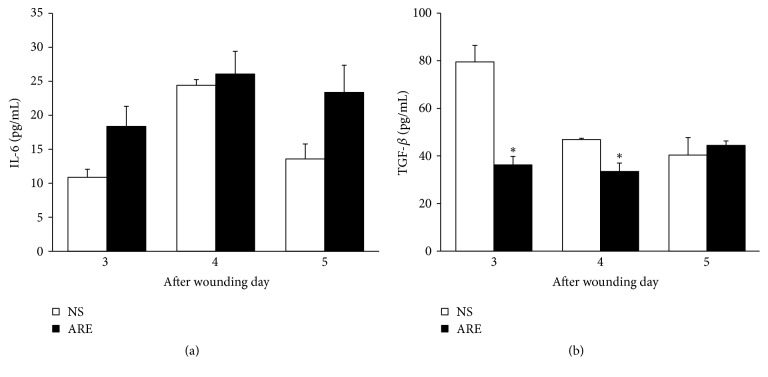
Plasma cytokine concentrations of (a) IL-6 and (b) TGF-*β* on days 3, 4, and 5, treated with NS and ARE. Measured by the enzyme-linked immunosorbent assay (ELISA). Data are expressed as mean ± standard error of the mean; ^*∗*^*P* < 0.05 indicates ARE group compared with NS group.

The levels of inflammatory cytokine IL-6 and the angiogenesis factor TGF-*β*1 at days 3–5 were measured to investigate treatment effectiveness.  Figure 4(a) showed elevation in the plasma IL-6 levels of the ARE group (18.38 pg/mL at day 3; 26.08 pg/mL at day 4; 23.38 pg/mL at day 5) exceeding that in the NS group (10.88 pg/mL at day 3; 24.42 pg/mL at day 4; 13.58 pg/mL at day 5) (*P* > 0.05). The plasma TGF-*β*1 levels of the ARE group were significantly lower than those in the NS group on days 3 and 4 (Figure 4(b)). On day 3, the TGF-*β*1 levels in NS and ARE groups were 79.52 pg/mL and 36.24 pg/mL (*P* < 0.05), respectively, and 46.89 pg/mL and 33.47 pg/mL (*P* < 0.05) on day 4.

## 4. Discussion

The TCM extracts, AE, RE, and ARE were used on excisional wound treatment because these extracts have antimicrobial and anti-inflammatory activities (Table 1, Figure 1). Our data revealed that treatment with ARE accelerated the healing of excisional wounds in the early stage of the wound healing process. The wound healing process consists of four continuous and overlapping phases. The hemostasis phase starts immediately after injury, followed by the inflammation phase, which continues for approximately 10 days. Meanwhile, the proliferative phase starts on the third day after wounding and continues for approximately 2 weeks. Remodeling and scar maturation starts during the proliferative phase and continues for several months [2]. In our data, the wound contraction rate of the ARE group was significantly higher than that of the control group on days 3–10 (Figure 2). Since day 3 to day 10 fall in the inflammation and proliferation phases, these results therefore suggest that the wound healing effect of ARE occurred within these phases.

Because the wound contraction of the ARE group was significantly lower than that of the NS group on days 3–5, we investigated histological changes in the wound healing process during this period. Collagen fibers play key components in wound healing, which provide structural support of skin strength [2]. Myofibroblasts are crucial cells involved in wound healing, which present more in the granulation tissue and disappeared after wound repair [23]. In our data, the histological observation revealed more collagen fibers and more myofibroblasts were present in ARE group than in NS group (Figure 3), suggesting that ARE treatment increases collagen fibers and myofibroblasts in the inflammation phase of wound healing process. The inflammatory cells, such as neutrophils and monocytes, are recruited to the wound site after injury [24]. Our histological characterization revealed that infiltration of inflammatory cell was more in ARE treated group than in NS group, suggesting ARE treatment increases inflammatory cell infiltration at the wound site during the inflammation phase.

The levels of inflammatory cytokine IL-6 and the angiogenesis factor TGF-*β*1 at days 3–5 were measured to investigate treatment effectiveness. Several studies have reported that IL-6 plays an influential role in wound healing. Kuhn et al. found that IL-6 is necessary for the efficient stimulation of epithelial proliferation after injury [25], while Lin et al. demonstrated that IL-6 knockout mice had impaired wound healing and suggested that IL-6 has crucial roles in wound healing, probably by regulating leukocyte infiltration, angiogenesis, and collagen accumulation [26]. Our results showed that the plasma IL-6 levels in the ARE group increased from day 3 to day 5 (Figure 4(a)), suggesting that wound contraction improved greatly in the ARE group during this period, possibly because of the increase in amount of IL-6. TGF-*β*1 reportedly plays an intricate role in modulating inflammation and immunity. Ashcroft et al. found that mice lacking Smad3, proteins that transduce signals from TGF-*β*1, exhibited accelerated wound healing and reduced inflammatory responses compared with those who had the proteins [27]. Li et al. found that overexpression of TGF-*β*1 in keratinocytes, a predominant cell type in the epidermis, causes chronic inflammation and delays wound healing [28]. However, Tang et al. reported that TGF-*β*1 mRNA expression was increased in emodin treatment group in wound tissue on day 7 [19]. We supposed that the opposite results of TGF-*β*1 were because these experiments were carried out on different days in wound healing process. The period of days 3-4 is involved in the inflammatory phase, while day 7 is involved in the proliferation phase [2]. Our data revealed that the plasma TGF-*β*1 levels of the ARE group were significantly lower than those in the NS group on days 3 and 4 (Figure 4(b)), indicating the lower levels of TGF-*β*1 accelerated wound healing process of inflammatory stage.

In AE, the active compound, furanocoumarins, possessed anti-inflammatory effect in the LPS-stimulated RAW264.7 macrophage cells [29]. Imperatorin, another active compound in AE, showed antioxidant effect protected renal injury by reducing NADPH oxidase and inhibiting of MAPK pathway [30]. AE can accelerate the proliferation of human keratinocytes and stimulate the cell cycle process of human keratinocytes by downregulation of cyclin D1 expression, thus enhancing the wound healing process [20]. In RE, the active compound, emodin, possessed anti-inflammatory activity via inhibiting the NF-*κ*B and MAPK signaling pathway [31], had antimicrobial effect against* S. aureus *[32], and enhanced wound healing in rats [19]. Rhein, another active compound found in RE, possessed antimicrobial effect against* S. aureus *[32] and protected endothelial cells against oxidative injury [33]. The antibacterial, anti-inflammatory, and antioxidant properties were related to the improvement of wound healing [34], suggesting that the effects of wound healing by ARE treatment may be attributed to the antibacterial, anti-inflammatory, and antioxidant activities in ARE. Comparing to a previous study in wound healing with emodin [19], our ARE treatment is much more effective on wound contraction, suggesting that, in addition to emodin, other active constituents, such as furanocoumarins, imperatorin, and rhein in ARE, may have contributed to the wound healing effects. Furthermore, our results showed that the healing effectiveness observed in ARE treated group was better than those in AE- or RE-treated groups, suggesting the synergistic effects of ARE on wound healing.

## 5. Conclusion

In this study, we demonstrated that ARE accelerates wound healing during the inflammation and proliferation phases. The present study suggests that ARE may be useful for application in wound treatment in the early stage of wound healing. We propose that ARE may have multiple effects in the treatment of skin lesions, including anti-inflammatory activity, antimicrobial activity, cell stimulation properties, and the promotion of wound healing.

## Figures and Tables

**Table 1:** Antimicrobial effect of AE, RE, and ARE.

TCM extracts	Zone of inhibition (mm)
AE	6.17 ± 0.17
RE	17.83 ± 0.60
ARE	15.50 ± 0.76
Blank	0
